# Exposure-shaped immunometabolic networks in chronic liver disease: translating multi-omics and artificial intelligence into preventive biomarkers

**DOI:** 10.3389/fimmu.2026.1866951

**Published:** 2026-09-22

**Authors:** Hailin Wang, Qinqin Tang, Juan Hu, Jingdong Li, Qiushi Huang

**Affiliations:** 1 Sichuan Clinical Research Center for Digestive Diseases, Affiliated Hospital of North Sichuan Medical College, Nanchong, China; 2 Department of Clinical Laboratory, Suining Central Hospital, Suining, China; 3 Department of Clinical Laboratory, Suining First People’s Hospital, Suining, China

**Keywords:** artificial intelligence, chronic liver disease, environmental exposures, exposome, immunometabolic networks, multi-omics, precision prevention, preventive biomarkers

## Abstract

Chronic liver disease develops through sustained interactions among metabolic stress, environmental exposure, immune activation, and tissue remodeling. These processes are often studied as separate domains, yet in patients they converge within the same hepatic microenvironment and help explain why disease trajectories vary even within the same diagnostic category. Exposure science has sharpened attention to diet, alcohol, pollutants, chemical mixtures, gut-derived signals, sleep and circadian disruption, and other behavioral determinants of liver injury. At the same time, multi-omics approaches now capture complementary dimensions of disease biology, including genetic susceptibility, epigenetic memory, inflammatory transcriptional programs, proteomic signaling, metabolic rewiring, microbiome composition, and spatially restricted cell states. The central challenge is no longer simply to generate more data, but to connect these layers into clinically useful markers of progression. In this review, we discuss chronic liver disease as a set of exposure-shaped immunometabolic network states that extend across steatosis, inflammation, fibrosis, cirrhosis, and hepatocellular transformation. We summarize how major exposure domains feed into shared pathogenic hubs, how immune and metabolic circuits sustain injury, and how genomics, epigenomics, transcriptomics, proteomics, metabolomics, microbiome profiling, and single-cell or spatial methods can reveal biologically coherent biomarker candidates. We also examine how machine learning, deep learning, network-based modeling, and causal-inference strategies may support risk stratification and progression forecasting when used with attention to interpretability, cohort structure, and external validation. A prevention-oriented biomarker framework should identify transition-prone states early enough to guide monitoring, referral, treatment selection, or lifestyle intervention. Such translation will require better exposure assessment, longitudinal sampling, assay standardization, and transportable models tested across real-world populations.

## 1 Introduction

Chronic liver disease (CLD) is a major and growing health burden that includes steatotic liver disease, alcohol-related liver injury, chronic inflammatory and fibrotic remodeling, cirrhosis, and hepatocellular transformation (1). These conditions are usually organized by dominant etiology, but clinical behavior often refuses to follow those labels. Patients with apparently similar diagnoses may differ widely in inflammatory activity, fibrotic progression, treatment response, and long-term outcome (2). That variability points to a broader biology: CLD is shaped by host susceptibility, environmental exposure, metabolic stress, immune dysregulation, and tissue repair programs acting together over time. Liver exposome research has made this point increasingly difficult to ignore. Environmental determinants are not merely background modifiers of liver health; in many settings, they participate in disease initiation, amplification, and phenotypic diversity (3).

The liver is especially exposed to this pressure because of its position between the external environment and internal metabolic homeostasis (3, 4). It senses nutrients, metabolizes xenobiotics, coordinates systemic energy balance, and filters gut-derived microbial products. It also receives signals from alcohol use, medication and toxicant exposure, air pollution, dietary pattern, sedentary behavior, sleep disruption, and circadian misalignment (5–7). These inputs differ in source and timing, but they often converge on overlapping biological programs: oxidative stress, lipid toxicity, mitochondrial dysfunction, inflammatory signaling, stromal activation, and maladaptive repair (8, 9). Over time, repeated exposure can reshape immune and metabolic circuits in ways that favor persistent injury. This makes CLD a useful setting for exposome science, because the liver does not merely register exposure; it biologically processes it.

Multi-omics technologies have changed the way liver disease is studied (10). Genomics and epigenomics describe inherited susceptibility and regulatory memory. Transcriptomics and proteomics capture active inflammatory and stress-response states. Metabolomics, lipidomics, microbiome profiling, single-cell sequencing, and spatially resolved approaches add information about biochemical flux, cell-state diversity, and tissue organization (11). Yet much of this information remains platform-centered. Studies often analyze one layer at a time, so connected processes are reported as separate findings. The result is an expanding literature that is rich in molecular detail but less successful at reconstructing how environmental stress becomes a disease-driving program or a clinically useful biomarker (12). Recent hepatology reviews have noted the promise of AI and multi-omics, but they also show how fragmented the field remains (13).

The missing step is not simply another dataset. What is needed is a model that links exposure history, immune-metabolic remodeling, and molecular readouts across disease stages. We use the term exposure-shaped immunometabolic network to describe a context-dependent disease state in which external and internal exposures are translated through hepatocyte stress, innate immune sensing, metabolic rewiring, stromal activation, and gut-liver signaling into measurable molecular programs. This framing differs from general systems-hepatology or immunometabolism models because exposure timing, mixtures, and biological processing are treated as upstream determinants of network-state formation and biomarker interpretation. Artificial intelligence can help interrogate such multidimensional data (14), but prediction alone is too narrow a goal. The stronger use case is the recovery of interpretable, prevention-oriented biological states.

Here, we develop this exposure-immunometabolic view of CLD and consider how it can inform biomarker discovery. Environmental exposures are treated as drivers of heterogeneity; immune and metabolic remodeling are considered the mechanistic bridge between exposure and progression; and multi-omics together with AI are discussed as tools for decoding these processes into biologically plausible and clinically useful markers. We use preventive biomarker to mean a marker that identifies transition-prone disease states, forecasts clinically meaningful progression, or guides earlier intervention, rather than simply describing established histologic damage. The review first examines major exposure domains, then follows their convergence on immune-metabolic networks across the CLD continuum. We then discuss how omics layers and AI-based integration can support early detection, risk stratification, and progression forecasting.

## 2 Environmental exposures and liver disease heterogeneity

Etiologic labels remain clinically useful, but they do not fully explain disease heterogeneity (15). Metabolic overload, alcohol, pollutants, chemical mixtures, gut-derived microbial signals, and circadian disruption can all feed into a limited set of pathogenic hubs, including lipotoxicity, mitochondrial dysfunction, oxidative stress, innate immune activation, and stellate-cell-driven fibrogenic remodeling (16–19). The strength of evidence differs across exposure classes. Metabolic overload and alcohol are supported by extensive mechanistic and clinical data, whereas pollutant mixtures and circadian disruption rely more heavily on observational cohorts, exposure proxies, and emerging mechanistic studies. Even so, these domains are relevant because they help explain why patients with the same diagnostic label may occupy different inflammatory, metabolic, and fibrotic states (20) (**Figure 1**).

**Figure 1:**
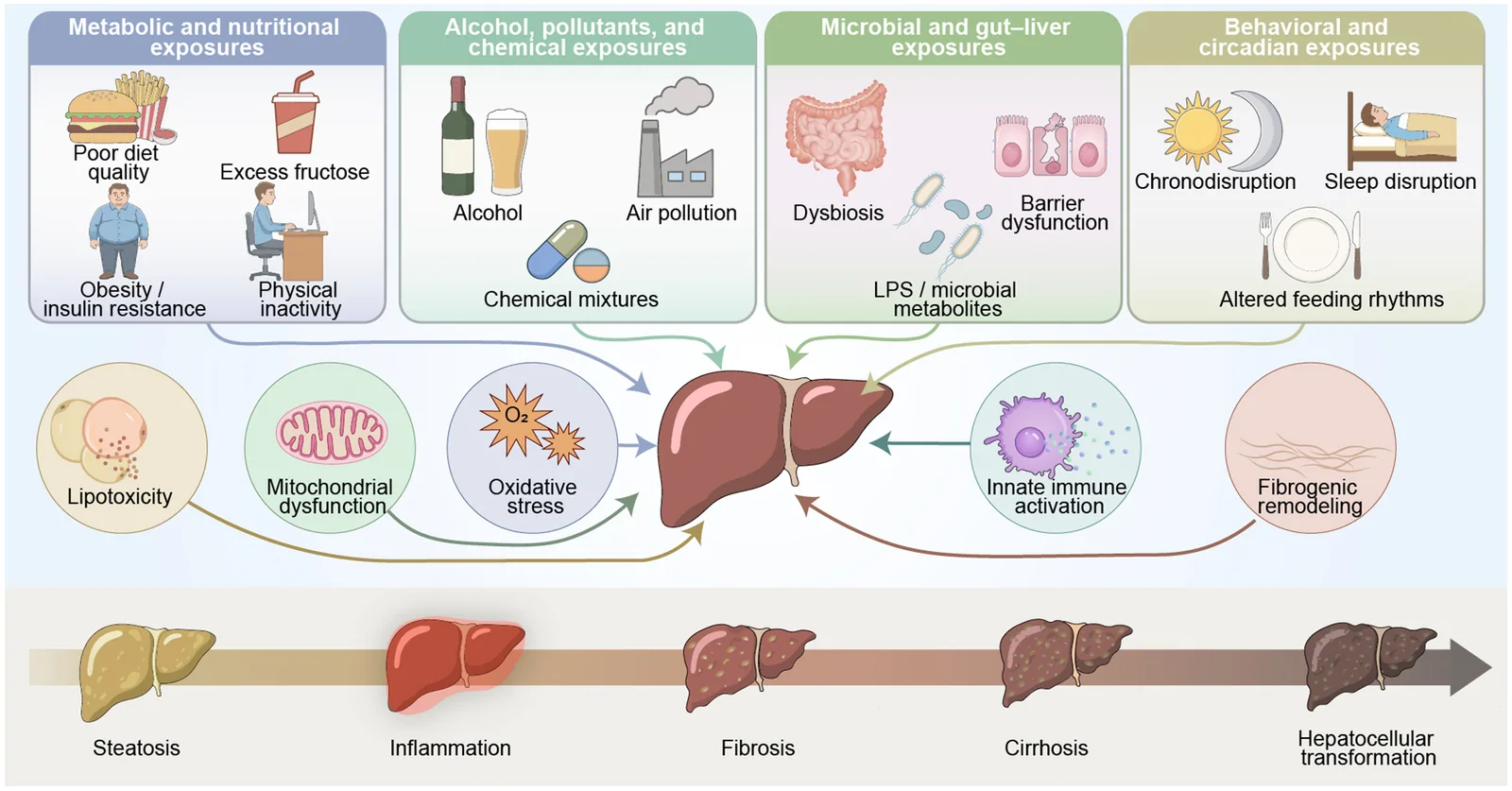
Exposure architecture driving chronic liver disease heterogeneity. Major exposure domains converge on shared pathogenic hubs, including lipotoxicity, mitochondrial dysfunction, oxidative stress, innate immune activation, and fibrogenic remodeling. Differences in exposure pattern and host context help shape heterogeneous trajectories across the CLD continuum.

### 2.1 Metabolic and nutritional exposures

Metabolic and nutritional stress is the dominant exposure axis in contemporary CLD, particularly within the MASLD spectrum (21). The current literature no longer treats this axis as simple lipid storage. Progression is linked to toxic lipid species, ER stress, mitochondrial dysfunction, ROS generation, JNK and NF-κB signaling, inflammasome activity, and insulin-resistant nutrient flux (22). Steatosis alone is therefore an incomplete descriptor. Recent mechanistic work increasingly places these pathways within nutrient-sensing and lipid-sensing circuits rather than isolated stress responses. Two patients may have similar hepatic fat content but differ substantially in inflammatory activity and fibrogenic potential, depending on the degree to which lipotoxicity, redox imbalance, and stress signaling are engaged (16). Recurrent nodes include SREBP1c, ChREBP, PPARα, AMPK, mTOR, JNK, NF-κB, and NLRP3, together with downstream cytokines such as TNF-α, IL-6, and IL-1β (22, 23).

Nutrition is not a single exposure. Excess fructose tends to amplify *de novo* lipogenesis and oxidative stress (24). Obesity and insulin resistance increase adipose lipolysis and hepatic fatty-acid influx (25). Reduced physical activity can worsen mitochondrial efficiency and systemic inflammatory tone, while poor dietary quality alters gut microbial composition and bile-acid pools. These inputs overlap, but they are not interchangeable. Their convergence on related yet distinct immunometabolic programs helps explain why the clinical label of metabolic liver disease covers a biologically diverse set of progression routes.

### 2.2 Alcohol, pollutants, and chemical exposures

Alcohol remains a distinct hepatotoxic exposure, but it is best interpreted within a broader toxicant framework rather than as a fully isolated category (26). Alcohol-associated liver injury reflects ethanol dose, acetaldehyde adduct formation, CYP2E1-dependent ROS generation, mitochondrial damage, gut barrier dysfunction, endotoxemia, and macrophage-mediated inflammatory amplification (27–29). Several of these processes overlap with metabolic liver injury, although alcohol places particular weight on oxidative metabolism, intestinal permeability, and innate immune activation (27). This overlap helps explain the aggressiveness of mixed metabolic-alcohol exposure states and the limitations of assigning liver injury to a single nominal etiology.

Non-alcohol chemical exposures require a measured interpretation. Ambient particulate matter, persistent organic pollutants, heavy metals, endocrine-disrupting chemicals, occupational exposures, and selected dietary or pharmacologic toxicants may contribute to liver injury through xenobiotic metabolism, oxidative stress, mitochondrial dysfunction, endocrine signaling, and inflammatory priming (7, 17, 19). The human evidence, however, is often observational and vulnerable to exposure misclassification, co-exposure confounding, and incomplete assessment of timing or cumulative dose. These limitations do not diminish the exposome concept. They define the methodological bar that pollutant-associated biomarker studies must meet.

### 2.3 Microbial and gut–liver exposures

The gut-liver axis is one of the clearest routes by which exposure is converted into hepatic biology (30). Through portal circulation, the liver receives microbial products, bile-acid derivatives, and microbial metabolites (30). Dysbiosis, barrier dysfunction, bacterial translocation, and altered microbial mediator profiles have been linked to MASLD, alcohol-associated liver disease, fibrosis progression, cirrhosis, and hepatocarcinogenic risk (31). The best-established mechanistic nodes include LPS-TLR4 signaling, inflammasome activation, bile-acid sensing through FXR and TGR5, short-chain fatty acid imbalance, and microbiota-dependent modulation of immune-cell recruitment and stellate-cell activation (30, 32). These microbial products can shape heterogeneity because they alter hepatocyte metabolism, immune-cell recruitment, bile-acid receptor signaling, and stromal activation in different combinations across patients.

Microbiome findings should be interpreted according to their role in the study design. A microbial feature may be an exposure readout shaped by diet, alcohol, medication, pollutants, and host context. It may also act as a mediator, a susceptibility modifier, or a biomarker summarizing host-microbe interaction. Treating these roles as equivalent can lead to overinterpretation. For biomarker development, the most useful microbiome signatures will be those connected to defined host pathways, reproducible clinical endpoints, and longitudinal change, rather than to taxonomy alone.

The microbial dimension is not secondary to liver injury (31). Diet, alcohol, and pollutants can reshape the intestinal ecosystem and barrier function, while microbiota-derived signals feed back into Kupffer-cell activation, cytokine release, bile-acid signaling, and fibrogenic crosstalk (30, 33). The same external exposure may therefore produce different hepatic consequences depending on how it is processed through the gut-liver interface. This is one reason substantial heterogeneity persists even within a single etiologic category.

### 2.4 Behavioral and circadian exposures

Behavioral and circadian disruption is a less mature but increasingly plausible exposure domain in CLD (34). Human studies and recent reviews link shift work, poor sleep, artificial light at night, chronodisruption, and altered feeding-fasting rhythms with steatotic liver disease and metabolic dysfunction (35). The biological rationale is strong: the hepatic clock regulates nutrient sensing, lipid turnover, bile-acid cycling, mitochondrial activity, and inflammatory timing. Circadian misalignment can therefore reprogram pathways already central to metabolic and inflammatory liver injury (36). Commonly discussed regulators include BMAL1/CLOCK, REV-ERB, PPAR signaling, rhythmic insulin sensitivity, and clock-controlled inflammatory-metabolic coupling (36).

The main value of this domain is its temporal structure. Circadian and behavioral exposures fluctuate over time, and their timing may alter the hepatic response to nutrients, toxicants, and microbial signals. A patient’s disease trajectory may be shaped not only by what exposures occur, but also by when and how they are organized. This makes behavioral timing difficult to measure, but it also makes it relevant to progression forecasting.

## 3 Exposure-shaped immunometabolic networks across the chronic liver disease continuum

Most exposure effects in CLD are not linear one-node pathways (37). Lipotoxic stress, ethanol-derived injury, microbial products, and pollutant-associated damage are integrated through coupled immune and metabolic circuits involving hepatocytes, Kupffer cells, recruited macrophages, hepatic stellate cells, and sinusoidal endothelial cells. These circuits often converge on mitochondrial dysfunction, oxidative stress, inflammasome activation, cytokine and chemokine signaling, and profibrotic matrix programs. Their relative dominance, however, differs across exposure contexts and disease stages (38). Similar histologic labels can therefore conceal different inflammatory and fibrogenic states (**Figure 2**).

**Figure 2:**
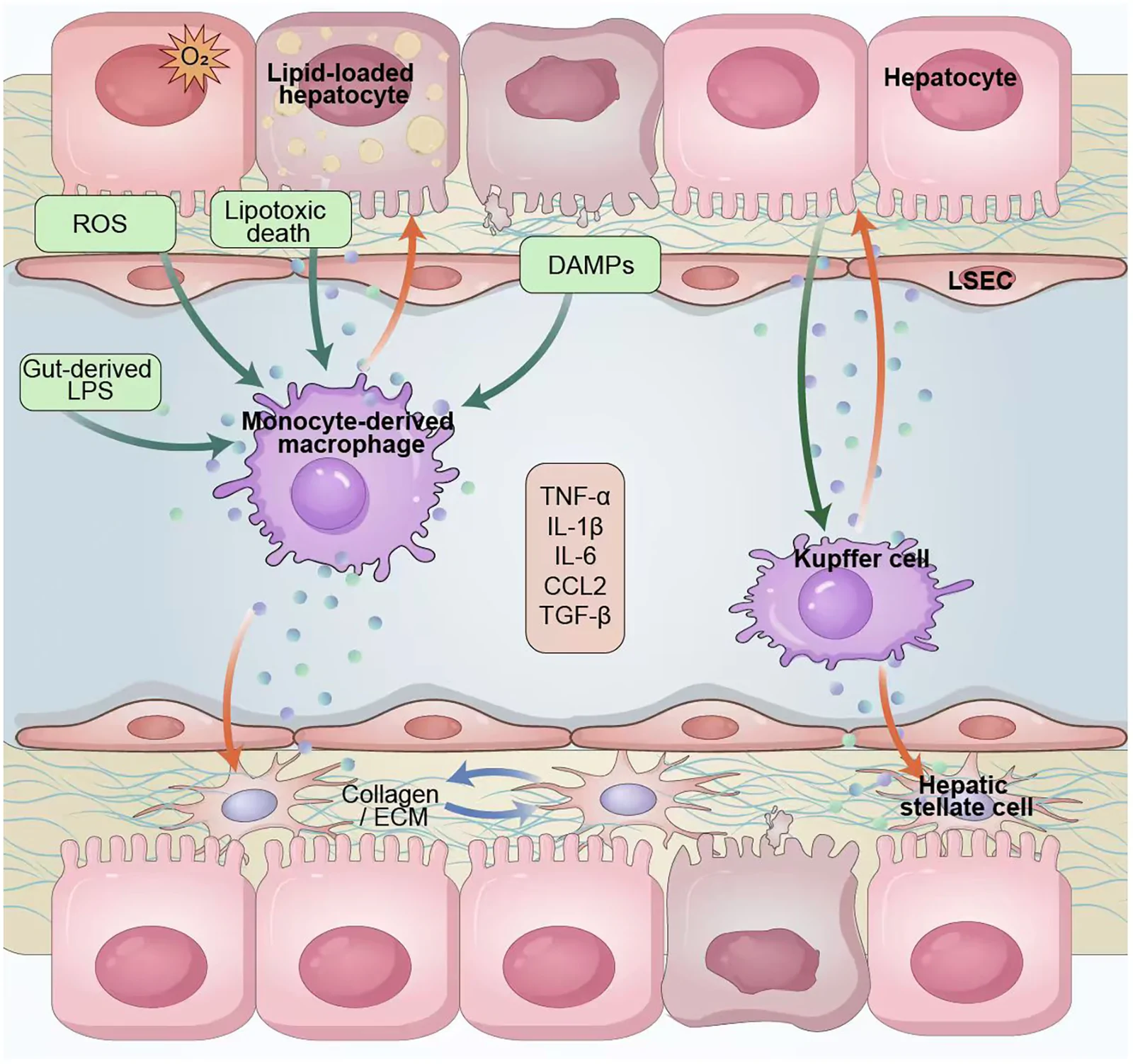
Exposure-associated injury sensing and inflammatory–fibrogenic crosstalk in the hepatic microenvironment. Hepatocyte injury signals and gut-derived inflammatory inputs activate macrophage-centered circuits that amplify hepatocyte stress and promote stellate-cell-associated collagen and extracellular matrix deposition.

### 3.1 Innate immune sensing and inflammatory amplification

Innate immune sensing is a major route through which environmental stress becomes persistent liver injury (39). In MASLD and related chronic liver states, Kupffer cells and monocyte-derived macrophages respond to lipotoxic hepatocyte death, ROS, damage-associated signals, gut-derived LPS, and xenobiotic stress. They then amplify injury through TNF-α, IL-1β, IL-6, CCL2, TGF-β, and other inflammatory mediators (39). Macrophages are central, but they are not the only sensing compartment: stressed hepatocytes, liver sinusoidal endothelial cells, cholangiocytes, neutrophil-associated responses, complement activity, and inflammasome-linked pathways can also participate in amplification. Recent macrophage-focused reviews also show why the older M1/M2 framework is inadequate in liver disease. Spatially and temporally heterogeneous macrophage states are more informative because distinct subsets can promote inflammation and stellate-cell activation, contribute to resolution, or participate in remodeling (40–42).

Macrophage biology illustrates why exposure history matters. The relevant issue is not simply whether macrophages are present, but how lipid overload, hypoxia, mitochondrial stress, inflammasome signaling, and gut-derived ligands shape their metabolism and crosstalk (38). These changes influence hepatocyte injury, neutrophil recruitment, and fibrogenic signaling. Innate immune sensing should therefore be viewed as an encoding system for exposure history, not only as a downstream reaction to tissue damage (43).

### 3.2 Metabolic rewiring as a driver of persistent liver injury

Metabolic reprogramming is not merely a passive consequence of inflammation; it is an active driver that sustains chronic liver injury and shapes immune-cell function. Immunometabolic studies in MASLD show that altered fatty-acid handling, mitochondrial dysfunction, defective mitophagy, redox imbalance, and nutrient-sensing abnormalities affect hepatocyte survival, macrophage activation, and stellate-cell behavior (44). Recurrently implicated pathways include AMPK, mTOR, PPAR signaling, JNK, NF-κB, NLRP3, and mitochondrial quality-control programs, together with changes in glycolysis, β-oxidation, and tricarboxylic-acid-linked metabolite flux (38).

Persistent liver injury emerges when metabolic stress and inflammation begin to reinforce one another. Hepatocyte lipotoxicity promotes inflammatory recruitment; inflammatory cytokines worsen insulin resistance and mitochondrial dysfunction; altered metabolite availability feeds back into macrophage and stellate-cell activation. This reciprocal structure helps explain why progression is often nonlinear and why a purely inflammatory or purely metabolic marker rarely captures the whole state.

### 3.3 Fibrogenic remodeling as sustained intercellular crosstalk

Fibrosis is often staged as a histologic endpoint, but biologically it reflects sustained intercellular communication (45). Hepatic stellate cells remain central to matrix remodeling, yet their activation is inseparable from inflammatory and metabolic crosstalk with macrophages, injured hepatocytes, endothelial cells, and gut-derived signals (46). TGF-β, PDGF, CCL2/CCR2-linked recruitment, inflammasome-associated mediators, angiocrine signaling, and extracellular matrix feedback loops all contribute to progressive stellate-cell activation and scar deposition (47, 48). In this sense, liver fibrosis reflects the stabilization of multicellular injury circuits and the cumulative output of immune-metabolic crosstalk.

Fibrosis-associated cell states also carry metabolic information. Stellate cells, macrophages, and other non-parenchymal cells adapt their glycolytic and mitochondrial programs during chronic injury, and these adaptations can stabilize inflammatory and profibrotic phenotypes. A fibrosis-associated signature may therefore represent more than collagen deposition; it can capture the accumulated output of immunometabolic crosstalk (49).

### 3.4 Network-state transitions across disease progression

The movement from steatosis to inflammation, fibrosis, cirrhosis, and hepatocarcinogenic transformation is better viewed as a sequence of network-state transitions than as a fixed ladder (49). Exposure patterns bias the liver toward different transition paths by altering innate immune sensing, metabolic adaptation, barrier function, cytokine circuitry, and stromal activation (38). In MASLD, for example, progression to MASH and advanced fibrosis reflects more than increasing fat content. It reflects a shift toward unstable immunometabolic states marked by amplified cell death, macrophage recruitment, inflammasome activity, and profibrotic communication (38). Similar principles apply in alcohol-related and mixed-exposure disease, where organ-to-organ and cell-to-cell crosstalk further diversify progression routes (37). Because these transitions are distributed across cell types, molecular layers, and tissue regions, they are difficult to resolve with a single marker or one platform. This provides the rationale for using multi-omics and AI-based integration to identify network states rather than isolated pathway fragments.

This view clarifies why conventional categories can underperform biologically (50). Two patients may meet criteria for the same disease stage while occupying different inflammatory-metabolic configurations and different risk states (51, 52). The clinically relevant unit is not only the exposure type or the diagnostic label, but the network state that links them.

## 4 Multi-omics decoding of exposure-associated liver pathology

Exposure-associated liver pathology is distributed across molecular layers rather than captured by any single platform (10). Each omics layer answers a different question. Genomics describes inherited susceptibility; epigenomics captures exposure-related regulatory memory; transcriptomics reports active injury and inflammatory programs; proteomics and metabolomics approximate functional output; microbiome profiling describes exposure processing through the gut-liver axis; and single-cell or spatial approaches localize these signals to the cell states and tissue niches in which they arise (10). This layered structure explains why patients with similar histology can still differ molecularly (53). Reviews of fibrosis, cirrhosis, and MASLD in the multi-omics era consistently support this interpretation.

### 4.1 Genomic and epigenomic layers

Genomics is best viewed as a susceptibility layer, while epigenomics is particularly relevant to exposure-linked state change because it can capture regulatory consequences of repeated metabolic, toxicant, inflammatory, or microbial pressure (54). In MASLD, human DNA methylation studies show that liver methylome remodeling tracks histologic severity, and recent methylome work has identified complement-related epigenetic alterations associated with disease severity rather than steatosis alone (55). Systematic reviews likewise support liver DNA methylation as a reproducible exposure-sensitive signal in metabolic liver disease. These findings matter because they suggest that epigenomic layers may preserve part of the regulatory history through which exposure becomes progression-prone biology.

Single-cell multiomics extends this point. Human liver studies integrating transcription, chromatin accessibility, histone marks, and three-dimensional chromatin structure show that disease-relevant regulatory programs cannot always be recovered from RNA alone. Cell-type-specific drivers of metabolic dysfunction may be missed by bulk approaches (56). Epigenomic data are therefore not decorative additions to transcriptomics; they help explain how exposure-related signals become stabilized in particular cell populations.

### 4.2 Transcriptomic layers

Bulk transcriptomics remains valuable because it captures inflammatory, stress-response, and matrix-remodeling programs at scale. Its main limitation is compositional averaging (10). Similar bulk signatures can arise from different mixtures of stressed hepatocytes, activated macrophages, stellate cells, and endothelial remodeling (53). For that reason, transcriptomic interpretation in CLD is moving from pathway abundance alone toward cell-state and tissue-context resolution. Reviews of liver fibrosis and cirrhosis increasingly make this distinction.

Human MASLD studies illustrate the shift. Spatially resolved multi-omics integrating single-cell gene expression with spatial transcriptomics has shown that metabolic dysfunction is organized through region-specific inflammatory and metabolic programs rather than uniform tissue-wide change (11). Zonation-focused work also shows that hepatocyte vulnerability and non-parenchymal responses differ across hepatic territories (57). For biomarker discovery, this is important because progression-associated transcriptomic states may be spatially restricted and cell-type specific (53).

### 4.3 Proteomic and metabolomic layers

Proteomic and metabolomic layers often sit closer to functional state than RNA alone (58). In advanced fibrosis and cirrhosis, integrative proteo-transcriptomic analyses indicate that clinically meaningful progression states are better defined when tissue transcriptomics is interpreted alongside circulating or tissue protein signals (59). Fibrosis-focused metabolomic studies have also identified reproducible shifts in amino-acid and phospholipid-related pathways, including histidine, choline, proline, glutamine, phenylalanine, tyrosine, citrate, and phosphocholine (60). These findings are not simply additional omics signals; they provide readouts of disease-stage biology.

Spatial multi-omics in MASLD reinforces this idea. Studies combining transcriptomics, proteomics, and mass-spectrometry-based spatial metabolomics have identified region-specific phospholipid abnormalities linked to tissue remodeling (61). Many exposures, including dietary overload, alcohol, pollutants, and microbiome-derived metabolites, first perturb flux, redox state, lipid handling, or bile-acid metabolism before they are visible histologically (58). Proteomic and metabolomic layers are therefore a practical bridge between upstream exposure and active liver injury (59).

### 4.4 Microbiome, single-cell, and spatial resolution

Because a substantial part of the exposome is processed through the gut-liver axis, microbiome profiling should not be treated as an optional add-on (62). A recent multi-omic human MASLD study described transkingdom gut dysbiosis, shifts in 66 bacterial species, enrichment of oral-type microbes, and coordinated host-microbiome alterations linked to liver dysfunction (63). Other host-microbiome metabolomic studies suggest that microbial and metabolic features together stratify MASLD-associated states better than host readouts alone (62). These findings support microbiome data as an exposure-processing layer, provided that interpretation remains tied to host biology.

Single-cell and spatial technologies add localization. They help determine where a candidate biomarker signal originates and whether it reflects a real tissue program or a bulk average (64). Human liver atlases and MASLD spatial multi-omics studies show that chronic injury is organized through zonation shifts, cell-neighborhood remodeling, and region-specific metabolic dysfunction (64). In practice, many useful biomarkers may be compressed readouts of a cell-state circuit, fibrogenic niche, or reproducible spatial injury program rather than single molecules in isolation (65, 66).

## 5 AI frameworks for exposome–multi-omics integration

AI is useful in CLD when it addresses a problem conventional analysis handles poorly: the relevant signal is distributed across exposure variables, clinical features, transcriptomic programs, regulatory states, metabolite and lipid profiles, microbiome composition, and spatially restricted cell states (14, 67). The goal should not be higher classification accuracy by itself. The more useful aim is to recover progression-relevant molecular states from complex, partially nonlinear data and convert them into biomarker panels that remain interpretable (**Figure 3**).

**Figure 3:**
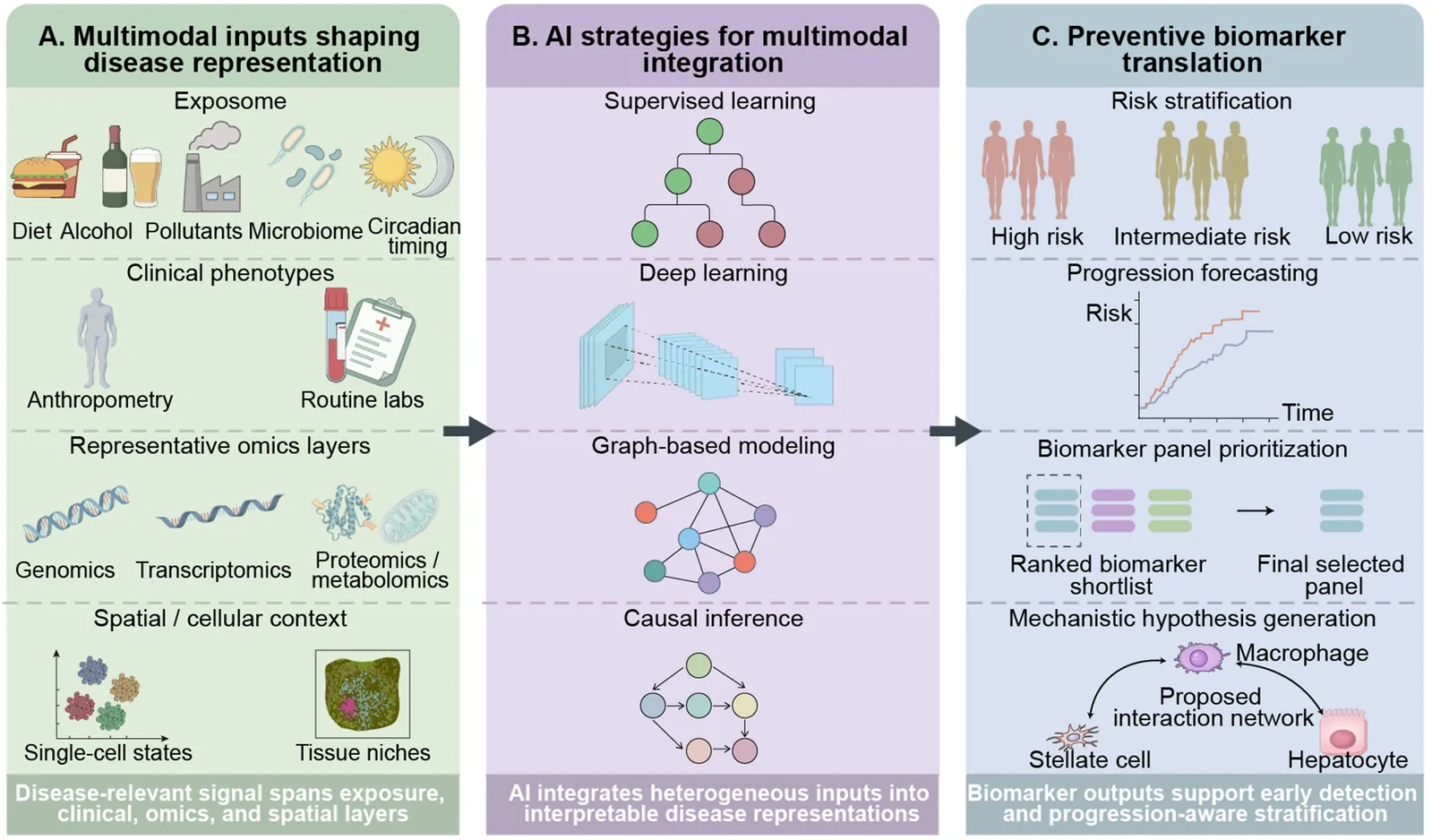
AI-enabled integration of exposome and multi-omics for preventive biomarker translation in chronic liver disease. Exposure, clinical, omics, and spatial data can be integrated through complementary AI strategies to support risk stratification, progression forecasting, biomarker prioritization, and mechanistic hypothesis generation.

Supervised learning remains the most mature application for biomarker prioritization and risk stratification (68). In MASLD/MASH, models built from mixed clinical variables have improved identification of high-risk steatohepatitis and significant fibrosis beyond conventional rule-based indices, suggesting that nonlinear interactions among routine features contain clinically relevant information (69–71). These approaches are valuable because they compress large candidate spaces into usable signatures for triage, enrichment, and risk modeling (69). Their weakness is also clear: performance often depends on cohort composition, assay platform, demographic structure, and background exposure pattern. Good internal discrimination does not guarantee external robustness (72).

Multimodal integration is more informative when it begins with biology rather than with model complexity (67). CLD progression is rarely encoded in one omics layer; it emerges from the joint behavior of inflammatory transcriptional programs, regulatory-state change, metabolic rewiring, host-microbiome interactions, and spatial remodeling (73). A MASLD-to-MASH study integrating bulk, single-cell, and spatial transcriptomic datasets with ensemble machine learning illustrates this point (73). The analysis resolved progression into a microenvironmental program centered on a DTNA-positive macrophage state, nominated RUNX2 as an upstream regulator, and proposed a RUNX2–PLG–PARD3 axis linking macrophages with activated stellate cells (73). In that setting, machine learning served as a prioritization tool after cell-state analysis, regulatory inference, and communication modeling had defined a plausible biological structure.

Network-aware methods are attractive because exposure-associated progression is propagated through coupled systems rather than isolated variables (74). Hepatocyte stress, macrophage activation, stellate-cell remodeling, bile-acid signaling, and gut-derived inflammatory inputs interact through dependency structures that flat feature rankings may miss (74, 75). Pathway-aware and graph-based strategies can preserve relationships among features and align better with immunometabolic disease architecture. Direct CLD applications remain limited, but the rationale is strong: if the biology is networked, the analytic strategy should not dissolve it into independent predictors. However, a network structure that improves prediction does not by itself establish causal direction or modifiability.

Causal inference is therefore also essential. Exposome-informed biomarker discovery cannot rely on association alone. Directed acyclic graphs can clarify confounding structures; mediation analysis can separate exposure effects transmitted through immune or metabolic intermediates; negative-control analyses can detect residual bias; Mendelian-randomization approaches may be useful where valid instruments exist; and target-trial emulation can strengthen longitudinal inference. These tools will not remove uncertainty, but they can help distinguish markers that track exposure background from markers that plausibly index modifiable disease mechanisms (76).

The main weakness of current AI studies in hepatology is limited external credibility, not insufficient model complexity (68). Many models are retrospective, internally validated, and population-specific. In an exposome-informed setting, this problem is amplified because environmental structure varies across cohorts, including diet, alcohol use, pollutant burden, microbiome ecology, and behavioral timing (77). A model trained in one exposure background may learn cohort-specific correlations rather than transportable disease biology (78). Explainability tools such as SHAP are helpful only when they show whether learned signals are biologically plausible and not dataset-specific artifacts (69). External validation, transportability, calibration, and biological coherence should carry as much weight as internal AUC.

Good modeling begins before model selection. Exposure variables require harmonized definitions, time windows, and units; omics layers require batch correction, normalization, and transparent feature selection; missingness should be modeled rather than quietly discarded; and calibration should be reported alongside discrimination. External testing should examine performance across centers, ancestry groups, metabolic backgrounds, alcohol patterns, pollutant burden, and microbiome ecology. In this context, a modest but interpretable and well-calibrated model may be more useful than a high-dimensional model that performs well only in its derivation cohort.

## 6 Preventive biomarker discovery and precision prevention

The key biomarker problem in CLD is not only identifying who already has steatohepatitis or advanced fibrosis. It is identifying who occupies a transition-prone state before irreversible remodeling or clinical decompensation dominates (79). That distinction separates preventive biomarkers from conventional diagnostic markers. Noninvasive tools are increasingly judged not only by how well they mirror biopsy stage, but by whether they identify at-risk MASH, significant fibrosis, future liver-related events, or impending decompensation early enough to change management (80).

In MASLD/MASH, the most established preventive use case is fibrosis-centered risk stratification (81). Blood-based scores and imaging-based noninvasive tests are already used to triage patients for specialist evaluation, treatment selection, and longitudinal monitoring because fibrosis stage remains the strongest predictor of liver-related outcomes (80, 82). Transient elastography, blood-based fibrosis panels, and sequential testing strategies are useful precisely because they move care upstream from biopsy-confirmed advanced disease toward earlier risk enrichment (82). They remain imperfect, however. Many tools perform better for advanced fibrosis than for inflammatory transition states, and accuracy can vary with metabolic context and population composition (83).

Omics-based biomarkers become relevant at this point. Their promise lies in identifying progression-associated states that precede overt cirrhosis or decompensation, including signatures linked to lipotoxic stress, macrophage activation, stellate-cell remodeling, bile-acid disturbance, and gut-liver dysregulation (84). Reviews of omics biomarkers in MASLD suggest that transcriptomic, proteomic, metabolomic, and methylation-derived panels may distinguish simple steatosis from inflammatory or fibrotic progression risk more effectively than conventional serum chemistry alone, especially when interpreted as coordinated signatures rather than isolated analytes (83). Most candidates still require external validation before they can be considered preventive tools in a clinical sense (83, 85).

Advanced CLD creates a second preventive use case: event prediction (86). In compensated cirrhosis, the relevant target is often not static fibrosis stage, but risk of portal hypertension, decompensation, hospitalization, or short-term mortality (86). Systematic reviews and prognostic biomarker studies suggest that inflammatory and injury-related markers such as IL-6, uNGAL, and copeptin may improve outcome prediction beyond traditional severity scores in selected settings (86–88). Prevention in hepatology therefore includes not only pre-fibrotic disease, but also prevention of first decompensation and other downstream events.

Current candidates are rarely ready for broad clinical use because many are developed within narrow biological or demographic contexts (89). A transcriptomic marker discovered in a metabolically dominant MASLD cohort may not behave similarly in populations with different alcohol exposure, pollutant burden, microbiome structure, or fibrosis prevalence (90). Precision-medicine reviews repeatedly emphasize that future biomarkers will need to integrate molecular state with clinical and exposure context rather than compete as single universal markers (91). The most credible path is likely sequential: broad risk enrichment first, progression-state refinement second, and longitudinal monitoring or treatment-response assessment third (92).

The field is moving beyond the question of whether one marker can replace biopsy in every context. A more realistic goal is a biomarker framework that identifies who is at risk, what type of progression state is present, and when intervention is most likely to alter trajectory. That is the practical meaning of precision prevention in CLD (**Table 1**).

**Table 1:** Exposure domains, immunometabolic mediators, omics readouts, and translational limitations relevant to preventive biomarker development in chronic liver disease.

Exposure domain	Dominant mediators	Informative omics layers	Preventive biomarker role	Current limitation
Metabolic and nutritional stress	Lipotoxicity, insulin-resistant nutrient flux, mitochondrial dysfunction, oxidative stress, inflammasome signaling	Metabolomics, lipidomics, transcriptomics, proteomics, epigenomics	Early enrichment of transition-prone MASLD/MASH states and fibrosis risk	Strong evidence base, but performance varies by obesity, diabetes, age, diet, and population structure
Alcohol and mixed metabolic-alcohol exposure	Acetaldehyde adducts, CYP2E1-related ROS, gut barrier dysfunction, endotoxemia, macrophage activation	Proteomics, metabolomics, microbiome profiling, inflammatory cytokine panels	Identification of mixed-exposure injury phenotypes and higher-risk progression trajectories	Exposure timing, drinking pattern, and coexisting metabolic disease are often incompletely captured
Pollutants and chemical mixtures	Xenobiotic metabolism, oxidative injury, endocrine disruption, mitochondrial stress, inflammatory priming	Exposomics, epigenomics, metabolomics, proteomics	Contextual modifiers of risk models and candidate markers of toxicant-associated susceptibility	Evidence is more heterogeneous and frequently observational; causal attribution remains difficult
Gut-liver and microbial signals	Dysbiosis, barrier dysfunction, LPS-TLR4 signaling, bile-acid signaling, microbial metabolites	Microbiome profiling, metabolomics, single-cell and spatial omics	Patient stratification by exposure-processing state and host-microbiome metabolic phenotype	Microbiome features can be exposure, mediator, modifier, or biomarker depending on study design
Behavioral and circadian disruption	Hepatic clock disruption, feeding-fasting misalignment, altered insulin and inflammatory rhythms	Time-stamped exposome measures, metabolomics, transcriptomics, wearable-derived phenotypes	Trajectory forecasting and identification of modifiable timing-related risk states	Evidence is promising but less mature; temporal exposure measurement is a major barrier

## 7 Discussion

An exposome-immunometabolic view helps explain why etiologic labels and single-platform biomarkers often underperform in CLD (3, 93). Environmental exposures are not interchangeable background risks. They are processed through partly overlapping but non-equivalent routes involving lipotoxicity, mitochondrial dysfunction, oxidative injury, gut-liver signaling, inflammatory amplification, and fibrogenic remodeling. This is the biological basis of heterogeneity: patients assigned to the same category may occupy different inflammatory, metabolic, and stromal states because their upstream exposure architecture differs (3).

Multi-omics adds value only when it resolves this heterogeneity into coherent biology rather than simply increasing dimensionality (94, 95). Genomic data define susceptibility; epigenomic layers retain regulatory consequences of sustained exposure; transcriptomic profiles capture active inflammatory and stress programs; proteomic and metabolomic data reflect functional output; and microbiome, single-cell, and spatial approaches show how these processes are distributed across the gut-liver axis, cell populations, and tissue niches (94). The strongest studies are not those with the most platforms, but those that show how progression-associated biology is organized across cells, regions, and modalities.

AI should be treated in the same way: useful, but not automatically informative (96). Its strongest role in CLD is not generic prediction, but recovery of stable, interpretable, and externally testable disease representations from heterogeneous exposure and omics data (97–99). Supervised models can enrich for high-risk steatohepatitis or fibrosis. Multimodal and network-based models may better recover inflammatory-metabolic states. Explainability tools are most useful when they test whether the learned signal remains biologically plausible (96, 97). The literature does not support the idea that more complex models alone will solve the biomarker problem.

The main translational gap is that many biomarkers still stage established disease better than they identify unstable transition states early enough to change trajectory (100). In MASLD, noninvasive tests already support fibrosis-centered triage and risk stratification, but they are less effective at resolving inflammatory transition states or exposure-defined subtypes (100). Blood-based and imaging-based fibrosis tools are clinically valuable, yet their performance varies with obesity, diabetes, age, and population background, and they remain more reliable for advanced fibrosis than for earlier progression biology (101).

A useful next step is not a single universal biomarker or a larger black-box model (102). It is a sequential, context-aware strategy that combines exposure information, omics-derived state markers, and clinically accessible noninvasive tests (102). Broad screening tools can remain useful for first-line enrichment. Deeper refinement of progression-prone states will probably require multimodal signatures that capture inflammatory, metabolic, microbial, and regulatory remodeling together (103). Longitudinal validation is essential because prevention-oriented hepatology depends on forecasting trajectory, not describing one time point (97). Operationally, high-dimensional discovery should be reduced through reproducible feature selection, pathway-level aggregation, assay feasibility checks, and external calibration into a minimal panel that can be measured alongside routine noninvasive tests.

This approach demands stronger study design than many current liver omics studies provide (3). Exposure assessment is often incomplete; mixtures are difficult to quantify; timing and duration are poorly modeled; and real-world populations differ in diet, alcohol patterning, pollutant burden, microbiome composition, and circadian disruption. These factors are not peripheral confounders. They help determine whether a biomarker is stable, transportable, or cohort-specific. CLD is therefore a demanding but informative test case for precision prevention because it sits at the intersection of metabolic disease, environmental burden, immune dysregulation, and long-term tissue remodeling.

For an exposure-aware preventive biomarker to be convincing, it should meet several conditions. It should be reproducibly associated with a prespecified clinical transition, add information beyond established clinical and noninvasive tests, remain calibrated across external cohorts, connect to plausible immune-metabolic mechanisms, and change a practical decision such as monitoring intensity, referral, treatment selection, lifestyle intervention, or response assessment (104, 105). Without that level of validation, a candidate marker may remain biologically interesting but not yet clinically preventive.
